# Supplementary material for: Molecular and cellular evidence for the impact of a hypertrophic cardiomyopathy-associated RAF1 variant on the structure and function of contractile machinery in bioartificial cardiac tissues
Source: Commun Biol. 2023 Jun 21;6:657. doi: 10.1038/s42003-023-05013-8 (PMC10284840; doi:10.1038/s42003-023-05013-8)
Supplement: Supplementary file 2 — Supplementary Information [file 42003_2023_5013_MOESM2_ESM.pdf]

Supplementary Information

Molecular and cellular evidence for the impact of a hypertrophy-associated RAF1 variant on the structure and function of contractile machinery in bioartificial cardiac tissues

Saeideh Nakhaei-Rad, Fereshteh Haghighi, Farhad Bazgir, Julia Dahlmann, Alexandra Viktoria Busley, Marcel Buchholzer, Karolin Kleemann, Anne Schänzer, Andrea Borchardt, Andreas Hahn, Sebastian Kötter, Denny Schanze, Ruchika Anand, Florian Funk, Annette Vera Kronenbitter, Jürgen Scheller, Roland P. Piekorz, Andreas S. Reichert, Marianne Volleth, Matthew J. Wolf, Ion Cristian Cirstea, Bruce D. Gelb, Marco Tartaglia, Joachim Schmitt, Martina Krüger, Ingo Kutschka, Lukas Cyganek, Martin Zenker, George Kensah, Mohammad R. Ahmadian

Institute of Biochemistry and Molecular Biology II, Medical Faculty and University Hospital Düsseldorf, Heinrich Heine University Düsseldorf, Düsseldorf, Germany

Table S1. The list of primers used in this study.

Name	Sequence	Accession number
Actinin alpha 1 (ACTN1)	CCCGAGCTGATTGACTACGG	NM_001130005.1
	GCAGTTCCAACGATGTCTTCG	
Alpha fetoprotein (AFP)	GAATGCTGCAAACTGACCACGCTGGAAC	NM_001354717.1
	TGGCATTCAAGAGGGTTTTCAGTCTGGA	
Actin beta (ACTB)	AGCCTCGCCTTTGCCGA	NM_001101.5
	CTGGTGCCTGGGGCG	
Calcium voltage-gated channel subunit alpha1 C (CACNA1C)	TGATTCCAACGCCACCAATTC	NM_199460.3
	GAGGAGTCCATAGGCGATTACT	
Calcium voltage-gated channel subunit alpha1 D (CACNA1D)	CGCGAACGAGGCAAACTATG	NM_001128840.2
	TTGGAGCTATTCGGCTGAGAA	
Troponin T2, cardiac type (TNNT2)	GGAGGAGTCCAAACCAAAGCC	NM_001001430
	TCAAAGTCCACTCTCTCTCCATC	
Forkhead box A2 (FOXA2)	TGGGAGCGGTGAAGATGGAAGGGCAC	NM_021784.4
	TCATGCCAGCGCCCACGTACGACGAC	
Hypoxanthine phosphoribosyl-transferase 1 (HPRT1)	CCTGGCGTCGTGATTAGTGAT	NM_000194.2
	AGACGTTCAGTCCTGTCCATAAT	
Kruppel like factor 4 (KLF4)	CAGCTTCACCTATCCGATCCG	NM_001314052.1
	GACTCCCTGCCATAGAGGAGG	
Myosin heavy chain 6 (MYH6)	GCCCTTTGACATTCGCACTG	NM_002471.3
	GGTTTCAGCAATGACCTTGCC	
Myosin heavy chain 6 (MYH7)	ACTGCCGAGACCGAGTATG	NM_000257.3
	GCGATCCTTGAGGTTGTAGAGC	
Myosin light chain (MYL2)	TTGGGCGAGTGAACGTGAAAA	NM_000432.3
	CCGAACGTAATCAGCCTTCAG	
Myosin light chain 7 (MYL7)	CATCAACTTCACCGTCTTCC	NM_021223.2

	GAAGCTGCTTGAACTCATCC	
Nanog homeobox (NANOG)	CCCCAGCCTTTACTCTTCCTA	NM_024865.3
	CCAGGTTGAATTGTTCCAGGTC	
Natriuretic peptide A (NPPA)	CAACGCAGACCTGATGGATTT	NM_006172.3
	AGCCCCCGCTTCTTCATTC	
Natriuretic peptide B (NPPB)	TCCTGCTCCTGCTCTTCTTG	NM_002521.2
	TCCTGTAACCCGGACGTTTC	
NK2 homeobox 5 (NKX2-5)	CCAGCCCTGCTCTCACG	NM_004387.4
	GCCCAGCGTAGGCCTCT	
POU class 5 homeobox 1 (POU5F1)/OCT4	CTGGGTTGATCCTCGGACCT	NM_002701.5
	CACAGAACTCATACGGCGGG	
Phospholamban (PLN)/PLB	ACCTCACTCGCTCAGCTATAA	NM_002667.4
	CATCACGATGATACAGATCAGCA	
Ryanodine receptor 2 (RYR2)	GGCAGCCCAAGGGTATCTC	NM_001035.2
	ACACAGCGCCACCTTCATAAT	
ATPase sarcoplasmic/endoplasmic reticulum Ca^{2+} transporting 2 (ATP2A2)/SERCA2	CATCAAGCACACTGATCCCGT	NM_001681.3
	CCACTCCCATAGCTTTCCCAG	
SRY-box 2 (SOX2)	GCCGAGTGGAAACTTTTGTCG	NM_003106.3
	GGCAGCGTGTACTTATCCTTCT	
SRY-box 17 (SOX17)	CGCTTTCATGGTGTGGGCTAAGGACG	NM_022454.4
	TAGTTGGGGTGGTCCTGCATGTGCTG	
Titin (TTN)	CCCCATCGCCCATAAGACAC	NM_133378
	CCACGTAGCCCTCTTGCTTC	
Troponin C1, slow skeletal and cardiac type (TNNC1)	TGGTTCGGTGCATGAAGGAC	NM_003280.2
	GTCGATGTAGCCATCAGCATTT	
Troponin I3, cardiac type (TNNI3)	AGAAGGAGGACACCGAGAAG	NM_000363.4
	GGAAGGCTCAGCTCTCAAAC	
Tubulin beta 3 class III (TUBB3)	ATGAGGGAGATCGTGCACAT	NM_006086.4
	GCCCCTGAGCGGACACTGT	
Skeletal muscle (ACTA1)/α-SK (ACTA1)	TGCCAACAACGTCATGTCG	NM_001100.3
	CAGCGCGGTGATCTCTTTCT	
Smooth muscle (ACTA2)/α-SMA	AAAAGACAGCTACGTGGGTGA	NM_001613
	GCCATGTTCTATCGGGTACTTC	
Titin (N2B isoform)	GGCCGAGAAATTTATGAGAGTGAC	NM_003319.4
	CGCTTTTCAGAACAACTTCTTCCT	
Titin (N2BA isoform)	CCAGCAACCAAGAAAGCTGCG	NM_001256850.1
	CCCAGAATCAGTTTTTGTGGTGTC	

Table S2. Reagents were used in this study.

p44/42 MAPK (total Erk 1/2) - Cell signaling #9102

Phospho-p44/42 MAPK (p-Erk1/2) (Thr202/Tyr204) - Cell signaling #4370

REAGENT	Concentration	SOURCE	IDENTIFIER
**Antibodies**			
anti-OCT3/4	1:200	Santa Cruz	#sc-5279
anti-cardiac troponin t	1:300	Thermo Fisher Scientific	#MA5-12960
anti-Tra-1-60	1:300	Abcam	#ab16288
anti-Myosin light chain 2 V	1:2000	Synaptic Systems	#310111
anti-SSEA4	1:70	DSHB	#MC-813-70
anti- γ -tubulin	1:1000	Sigma-Aldrich	#T5326
anti-phospho-ERK1/2 T202/T204	1:1000	Cell Signaling	#9106
anti-ERK1/2	1:1000	Cell Signaling	#9102
anti-phospho-AKT S473	1:1000	Cell Signaling	#4060
anti-phospho-AKT T308	1:1000	Cell Signaling	#2965
anti-AKT	1:1000	Cell Signaling	#9272
anti-phospho-YAP Ser127	1:500	Cell Signaling	#4911
anti-YAP	1:1000	Cell Signaling	#4912
anti-JNK	1:500	Cell Signaling	#9252
anti-phospho-JNK Thr183/Tyr185	1:200	Cell Signaling	#9251
anti-S6K	1:1000	Cell Signaling	#2708
anti-phospho-S6K Thr389	1:500	Cell Signaling	#9205
anti-phospho-p38 Thr180/Tyr182	1:1000	Cell Signaling	#9211
anti-p38	1:1000	Cell Signaling	#8690
anti-alpha-actinin	1:300	Sigma-Aldrich	#A7811
anti-ATP2A2/SERCA2	1:1000	Cell Signaling	#4388
anti-RAF1	1:150	Abcam	#AB181115
anti-phospho-RAF1 S259	1:500	Abcam	#ab173539
anti-TUBB3	1:1000	Thermo Fisher Scientific	#MA1-118
anti-Nkx2.5	1:25	Santa Cruz	#sc-14033
anti-Sox17	1:50	R&D Systems	#AF1924
anti-desmin	1:100	Agilent Technologies	#M076029-2
anti-troponin I	1:500	Abcam	#Ab47003
anti-SMA Clone 1A4	1:2000	DAKO	#M0851
anti-GAPDH	1:1000	Thermo Fisher Scientific	# 398600
anti-rabbit IgG Alexa Fluor 488 Conjugate	1:500	Cell Signaling	#4412
anti-mouse IgG Alexa Fluor 555 Conjugate	1:500	Cell Signaling	#4409
anti-mouse IgG Alexa Fluor 488 conjugated	1:500	Cell Signaling	#4408
IRDye® 800CW Donkey anti-Rabbit IgG	1:10000	LI-COR Biosciences	#926-32213
IRDye® 800CW Donkey anti-Mouse IgG	1:10000	LI-COR Biosciences	#926-32212
Alexa 488-conjugated goat anti-rabbit IgG	1:200	Thermo Fisher Scientific	#A11034
Alexa 546-conjugated goat anti-mouse IgG	1:200	Thermo Fisher Scientific	#A4671
Alexa 488-conjugated goat anti-mouse IgG	1:200	Thermo Fisher Scientific	#A11029
Custom made α-titin PEVK raised against PEVK S11878 (CEVVLKSVLRKR) and PEVK S12022 (LRPGSGGEKPP) (Kötter, Sebastian, et al. Circulation research, 2016)	1:200	Eurogentec	N/A
**Chemicals, peptides, and recombinant proteins**			
B-mercaptethanol		Sigma-Aldrich	#M-3148
basic Fibroblast Growth Factor (bFGF)		Peprotech	#100-18B
ROCK-Inhibitor Y27632		Selleckchem	#S1049
CHIR99021		Selleckchem	#S1263
IWR-1		Sigma-Aldrich	#I0161
RPMI 1640 Medium, no glucose		Thermo Fisher Scientific	#11879-020
human Albumin		Sigma-Aldrich	#A0237
Sodium DL-Lactat		Sigma-Aldrich	#L4263
L-Ascorbic acid-2-Phosphate		Sigma-Aldrich	#A8960

DMEM / F12 + Glutamax	Thermo Fisher Scientific	#31331-028
Knockout Serum Replacement (KO-SR)	Thermo Fisher Scientific	#10828028
MEM Non-Essential Amino Acids	Thermo Fisher Scientific	#11140-035
RPMI 1640 Medium	Thermo Fisher Scientific	#21875-034
B-27™ Supplement, minus insulin	Thermo Fisher Scientific	#A1895601
B-27™ Supplement	Thermo Fisher Scientific	#17504044
Geltrex Membrane Matrix	Thermo Fisher Scientific	#A1413201
Gelatine from porcine skin	Sigma-Aldrich	#G2500
Accutase™ Cell Dissociation Reagent	Thermo Fisher Scientific	#A1110501
Collagenase, Type IV	Thermo Fisher Scientific	#17104019
Agarose NEEO Ultra-Quality	CARL ROTH	#2267.4
Hydrosil A and Hydrosil B	SILADENT	#101301
AggreWell™400	STEMCELL Technologies	#27840
Versene Solution	Thermo Fisher Scientific	15040066
TrypLE™ Select Enzyme	Thermo Fisher Scientific	A1285901
DMSO (dimethyl sulfoxide)	Sigma-Aldrich	#D2650
Fetal Bovine Serum	Thermo Fisher Scientific	26140079
L-Glutamine	Thermo Fisher Scientific	25030149
KaryoMAX Colcemid	Thermo Fisher Scientific	15212012
Trypsin/EDTA	Biochrom	L 2143
TRIzol™	Thermo Fisher Scientific	15596026
SYBR™ Green	Applied Biosystems	#4309155
Formaldehyde 4%	Carl Roth	#P087.1
EDTA-free protease inhibitor	Sigma-Aldrich	11873580001
Phosphate buffered saline with 5% non-fat milk	Merck	#P4739
Intercept® (TBS) Blocking Buffer	LI-COR	927-60001
ProLong™ Gold Antifade Mountant	Thermo Fisher Scientific	P10144
Tissue-Tek® O.C.T. Compound	Sakura Finetek	#4583
**Cell Lines**		
HFF-1	ATCC	SCRC-1041
**Recombinant DNA**		
pCE-hSK	Addgene	#41814
pCE-hOct3/4	Addgene	#41813
pCE-hUL	Addgene	#41855
pCE-mp53DD	Addgene	#41856
pCXB-EBNA1	Addgene	#41857
**Oligonucleotides**		
For primers, please see Table S1		
**Software**		
FlowJo	Treestar, Ashland, OR	https://www.flowjo.com/
IKAROS	MetaSystems (Altlußheim, Germany)	https://metasystems-international.com/de/products/ikaros
Image Studio 5.2	LI-COR	https://www.licor.com/bio/image-studio-lite
ZEN 3.2 (blue edition)	Carl Zeiss AG	
IonWizard 6.4	Ion Optix Corp	http://www.ionoptix.com/
Prism 6	GraphPad software	https://www.graphpad.com/scientific-software/prism/

**Critical Commercial Assays**		
DNA-free™ DNA Removal Kit	Invitrogen	AM1906
GoScript™ cDNA Synthesis Kit	Promega	A5003
Quick Start™ Bradford Protein Assay	Bio-Rad	5000201
Trichrome II Blue staining kit	Roche	#860-013
**Other**		
6-Well-Plate	Thermo Fisher Scientific	140675
T-25 Tissue Culture Flasks	TPP Techno Plastic	90026
T-175 Filter Cap Flasks	CELLSTAR	GR661175

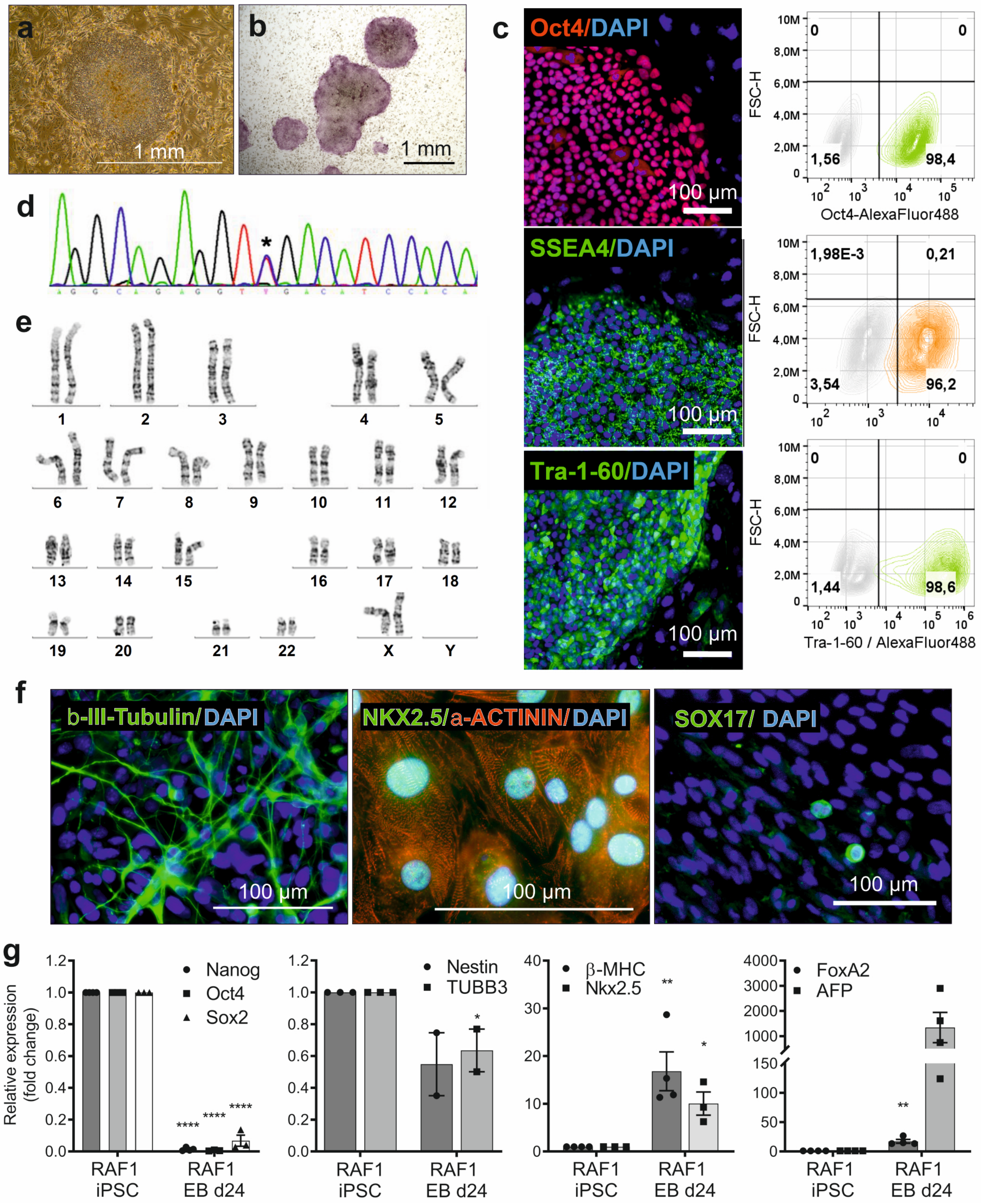

Figure S1. Characterization of RAF1^{S257L} iPSC line 1 (7B10) from the first patient. (a) Typical iPSC colonies on mitotically inactivated murine feeder cells. **(b)** Alkaline phosphatase-positive colonies. **(c)** Expression of pluripotency markers OCT4, SSEA4, and TRA-1-60 as detected by immunofluorescence staining and flow cytometry. Isotype controls of flow cytometry histograms are depicted in light gray. **(d)** Sanger sequencing confirmed the heterozygous *RAF1 c.770C>T* variant in iPSCs (asterisk). **(e)** Normal diploid karyotypes in iPSCs at passage 8 after reprogramming. **(f)** Trilineage differentiation of patient-derived iPSCs. Expression of ectodermal (beta-III-Tubulin), mesodermal (NKX2.5 and sarcomeric alpha-actinin), and endodermal (SOX17) markers was detected. **(g)** Relative gene expression of pluripotency (*NANOG*, *OCT4*, *SOX2*) and differentiation markers (*NESTIN*, *TUBB3*, *MYH7*, *NKX2.5*, *FOXA2*, *AFP*) of differentiated embryoid bodies on d24 of differentiation relative to

undifferentiated iPSCs normalized by beta-ACTIN expression. Bar graphs represent the mean of three independent samples +/- SEM. *P < 0.05, **P < 0.01, ***P < 0.001, ****P < 0.0001, unpaired t test. n=3, biological replicates. APF, alpha fetoprotein; EB, embryoid body; FOXA2, forkhead box A2; MYH, myosin heavy chain; NKX2.5, NK2 homeobox 5; TUBB3, tubulin beta 3 class III, WT, wild-type.

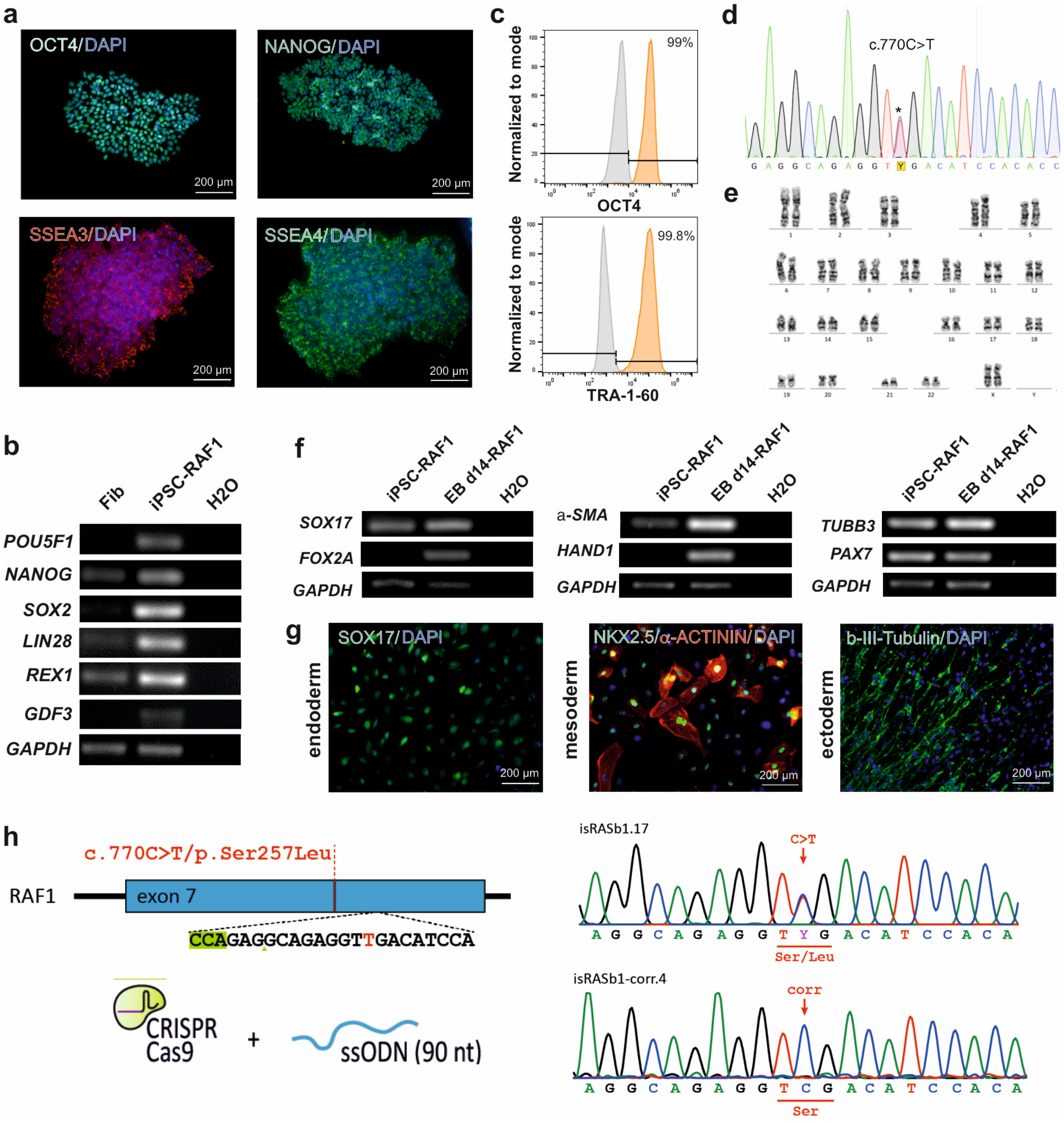

Figure S2. Characterization of RAF1^{S257L} iPSC line 2 (isRASb1.17) from the second patient. Human iPSC-RAF1^{S257L} line 2 reveals expression of pluripotency markers, a normal karyotype, and differentiation potency towards ectodermal, endodermal, and mesodermal derivatives *in vitro*. **(a)** iPSCs-RAF1^{S257L} line 2 stain positive for OCT4, NANOG, SSEA3 and SSEA4 as detected by immunofluorescence staining. **(b)** qPCR analysis of pluripotency markers in iPSCs and control fibroblasts. **(c)** Flow cytometry analysis confirmed more than 99% of iPSCs stain positive for OCT4 and TRA-1-60. **(d)** Sanger sequencing confirmed the heterozygous RAF1^{S257L} variant in iPSCs (asterisk). **(e)** iPSCs show a normal diploid karyotype. **(f, g)** Trilineage differentiation of iPSC-RAF1^{S257L}. Expression of endodermal (SOX17 and FOX2A), mesodermal (*α-SMA*, *HAND1*, NKX2.5, and sarcomeric alpha-actinin), and ectodermal (TUBB3 and *PAX7*), markers were detected by RT-PCR and immunofluorescence staining. **(h)** CRISPR-CAS9 mediated correction of the RAF1^{S257L} mutation to RAF1^{WT} for the line 2 iPSCs (isRASb1-corr). APF, alpha fetoprotein; EB, embryoid body; FOXA2, forkhead box A2; HAND1, Heart- and neural crest derivatives-expressed protein 1; NKX2.5, NK2 homeobox 5; PAX7, Paired Box 7; TUBB3, tubulin beta 3 class III.

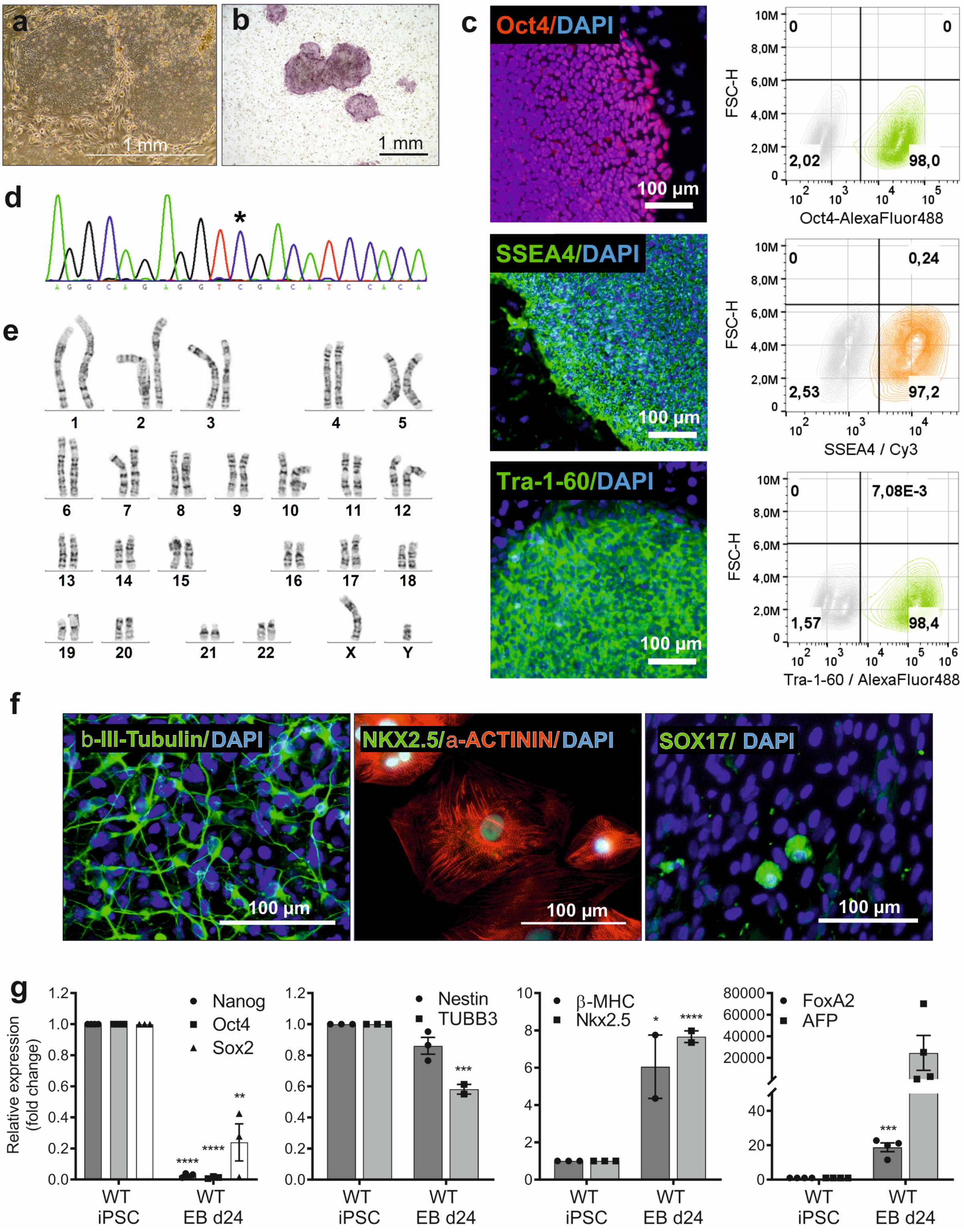

Figure S3. Characterization of WT iPSC line ipWT16.1 from donor 1 (WT1). (a) Typical iPSC colonies on mitotically inactivated murine feeder cells. **(b)** Alkaline phosphatase-positive colonies. **(c)** Expression of pluripotency markers OCT4, SSEA4, and TRA-1-60 as detected by immunofluorescence staining and flow cytometry. Isotype controls of flow cytometry histograms are depicted in light gray. **(d)** Sanger sequencing confirmed the wild-type RAF1 sequence in iPSCs (asterisk). **(e)** Normal diploid karyotypes in iPSCs at passage 8 after reprogramming. **(f)** Trilineage differentiation of patient-derived iPSCs. Expression of ectodermal (beta-III-Tubulin), mesodermal (NKX2.5 and sarcomeric alpha-actinin), and endodermal (SOX17) markers was detected. **(g)** Relative gene expression of pluripotency (*NANOG*, *OCT4*, *SOX2*) and differentiation markers (*NESTIN*, *TUBB3*, *MYH7*, *NKX2.5*,

FOXA2, *AFP*) of differentiated embryoid bodies on d24 of differentiation relative to undifferentiated iPSCs normalized by beta-actin expression. Bar graphs represent the mean of three independent samples +/- SEM. *P < 0.05, **P < 0.01, ***P < 0.001, ****P < 0.0001, unpaired t test, n=3, biological replicates. APF, alpha fetoprotein; EB, embryoid body; FOXA2, forkhead box A2; MYH, myosin heavy chain; NKX2.5, NK2 homeobox 5; TUBB3, tubulin beta 3 class III, WT, wild-type.

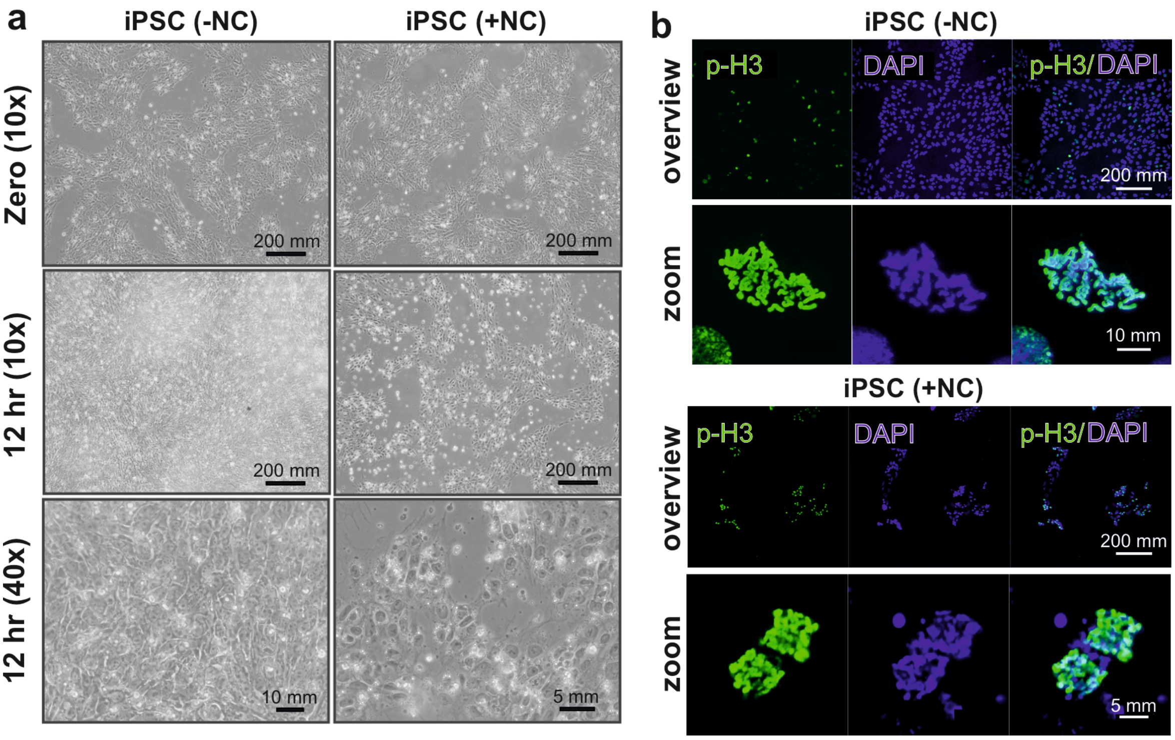

Figure S4. Morphology of the RAF1^{S257L} iPSC line 1 after Nocodazole (NC) treatment. (a) Illustration of cells arrested in mitosis upon NC treatment (100 nM) (lower panels). **(b)** phospho-histone 3 (p-H3) standing of human iPSC with different magnifications after NC treatment. cTNT, cardiac troponin T; iPSC, induced pluripotent stem cells; p-H3, phospho-histone 3; NC, Nocodazole.

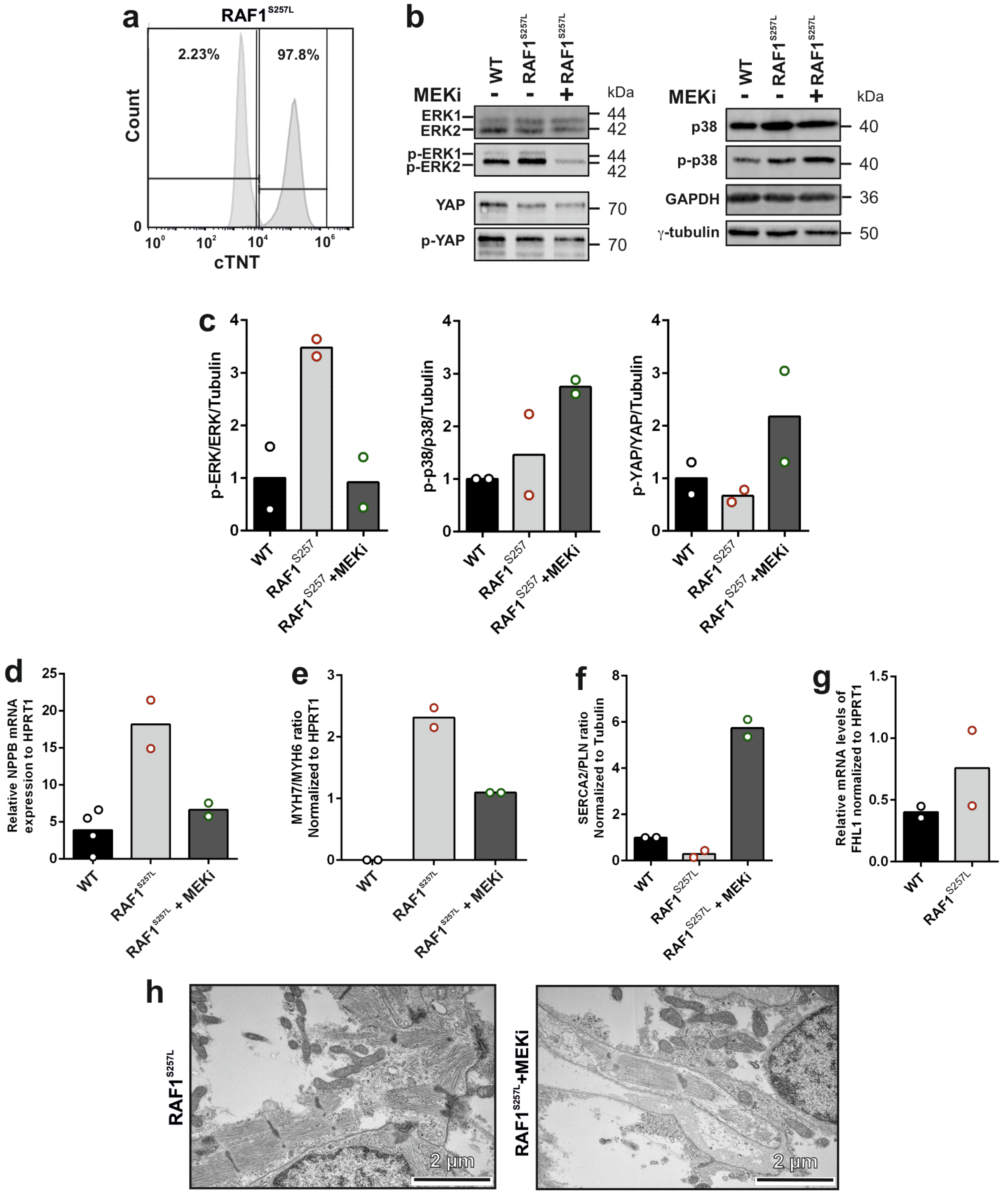

Figure S5. Analysis of cardiomyocytes generated from a second individual carrying RAF1S257L (iPSC line 2). **(a)** Expression of cardiac troponin C marker by flow cytometry at day 24 of cardiac differentiation. **(b)** Western blots analysis of the mentioned signaling molecules, p-ERK/ERK, p-YAP/YAP, and p-p38/p38. **(c)** Quantification of phospho-protein to total protein ratios. *P < 0.05, unpaired 1-tail t-test, n≥2, biological replicates. **(d)** Quantitative real-time PCR analysis of *NPPB* transcript levels. **P < 0.01, unpaired 2-tail t-test, n≥2, biological replicates. **(e)** *MYH7-to-MYH6* ratio was compared between WT-CBs and RAF1^{S257L} CBs according to $2^{(-\Delta ct)}$ values. **(f)** Quantification of western blot results of SERCA2/PLN protein ratio normalized to Tubulin. *P < 0.05, unpaired 2-tail t-test, n≥2, biological replicates. **(g)** Quantitative real-time PCR analysis of *FHL1* transcript levels, n≥2, technical replicates. **(h)** Electron microscopic images of RAF1^{S257L}-CBs treated with 0.2 μM MEK inhibitor from d12 of differentiation.

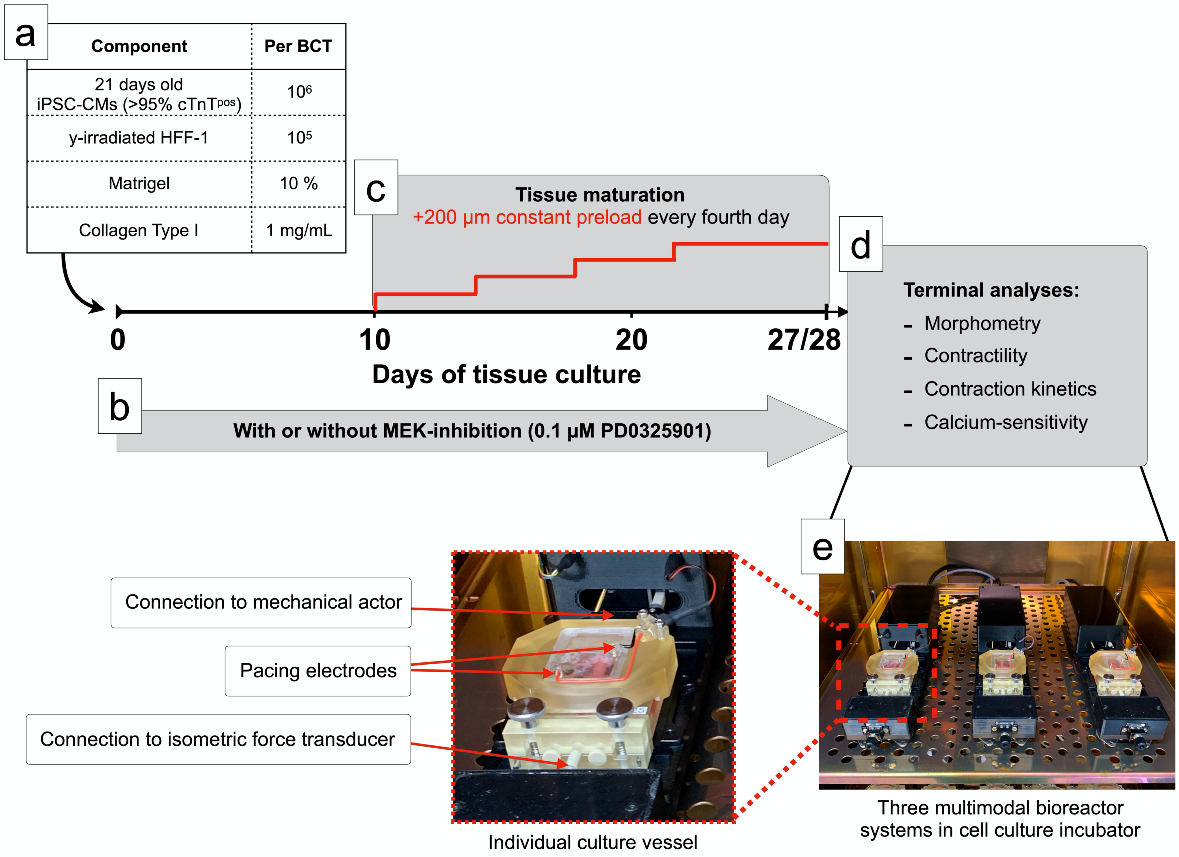

Figure S6. Overview of bioartificial cardiac tissue (BCT) generation, culture, treatment, and measurement. (a) One BCT (initial volume: 250 µL) is composed of one million purified iPSC-derived non-treated CMs (>95% $cTnT^{pos}$) of either genotype ($RAF1^{corr}$ and $RAF1^{S257L}$), 0.1 million γ-irradiated human foreskin fibroblasts and 10% Matrigel and 1 mg/mL rat tail collagen type I. **(b)** The culture medium of the treatment group of $RAF1^{S257L}$-BCTs was supplemented with 0.1 µM PD0325901 from day 0. **(c)** To support tissue maturation, BCTs were stretched by 200 µm increments every fourth day, starting on day 10 of tissue culture four times. **(d)** On day 27 or 28 of culture, tissue samples were subjected to terminal physiological analyses in multimodal bioreactor systems allowing for in-depth assessment of contractility, contractile kinetics, and calcium-sensitivity of BCTs **(e)**. Inset shows an individual culture vessel that is equipped with two platinum electrodes for field stimulation and which is connected to a motor to apply precise preload and to an isometric force transducer to record contraction forces.

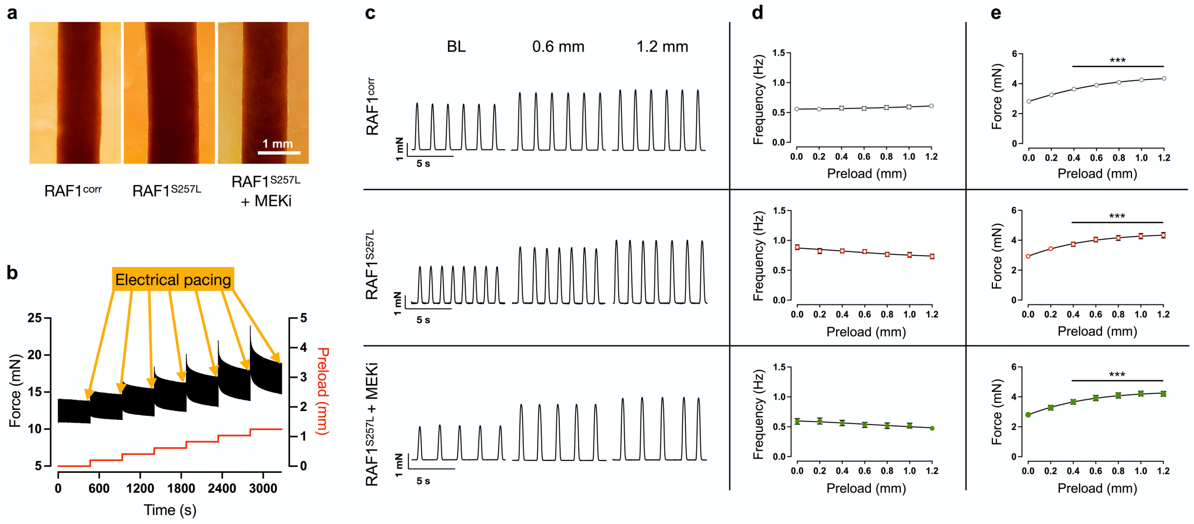

Figure S7. Preload-dependent behavior of BCT contractility. (a) Representative brightfield images of the three experimental groups before measurement on days 27 - 28 of culture. Scale bar: 1 mm. **(b)** Original traces of a representative measurement to determine the maximum contraction force of a BCT. Starting from a defined baseline length (BL) for all tissues, i.e. 6.8 mm, preload (red) is increased by 200 µm increments and the spontaneous contractions (black) are recorded constantly. Before each preload step, the BCT is electrically paced five times at a frequency slightly above its spontaneous beating frequency but never below 1 Hz (yellow arrows). **(c)** Representative original traces of spontaneous contractions for each experimental group were recorded at baseline (left), 0.6 mm (center), and 1.2 mm (right) preload, respectively. **(d)** Spontaneous contraction frequencies of BCTs in response to increasing preload. **(e)** Preload-dependent physiological increase in paced contraction forces. For D and E: n = 18-26 individual tissue samples per group. Two-way ANOVA was applied. Error bars: ± SEM. ***P<0.001 *vs*. baseline. Analysis of cardiomyocytes generated from the second individual carrying RAF1^{S257L} (iPSC line 2).

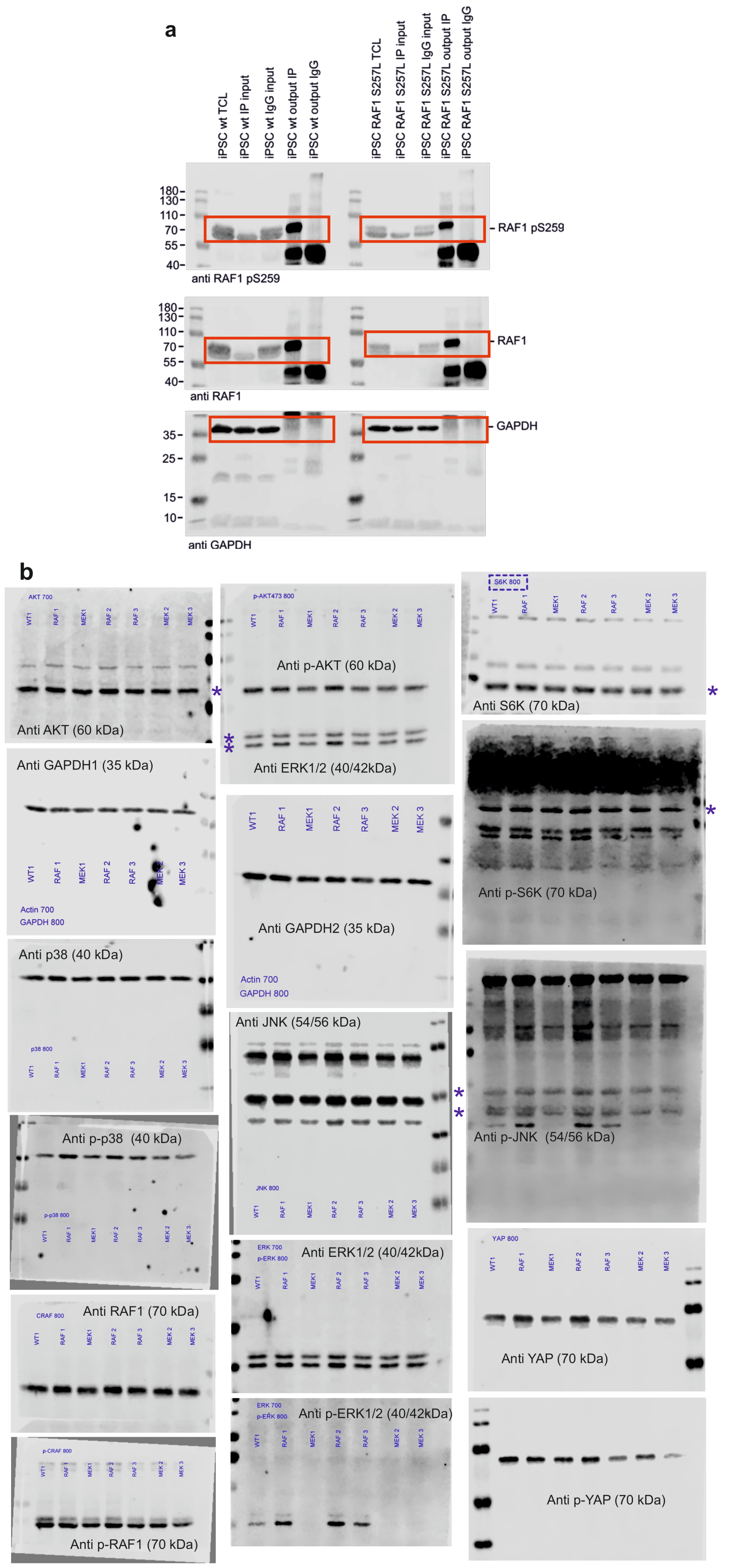
a
iPSC wt TCL
iPSC wt IP input
iPSC wt IgG input
iPSC wt output IP
iPSC wt output IgG
iPSC RAF1 S257L TCL
iPSC RAF1 S257L IP input
iPSC RAF1 S257L IgG input
iPSC RAF1 S257L output IP
iPSC RAF1 S257L output IgG
180
130
110
70
55
40
RAF1 pS259
anti RAF1 pS259
RAF1
anti RAF1
35
25
15
10
GAPDH
anti GAPDH
b
Anti AKT (60 kDa)
Anti p-AKT (60 kDa)
Anti ERK1/2 (40/42kDa)
Anti S6K (70 kDa)
Anti GAPDH1 (35 kDa)
Anti p-S6K (70 kDa)
Anti GAPDH2 (35 kDa)
Anti p38 (40 kDa)
Anti JNK (54/56 kDa)
Anti p-JNK (54/56 kDa)
Anti p-p38 (40 kDa)
Anti ERK1/2 (40/42kDa)
Anti RAF1 (70 kDa)
Anti YAP (70 kDa)
Anti p-ERK1/2 (40/42kDa)
Anti p-YAP (70 kDa)
Anti p-RAF1 (70 kDa)
WT1
RAF 1
MEK1
RAF 2
RAF 3
MEK 2
MEK 3
*

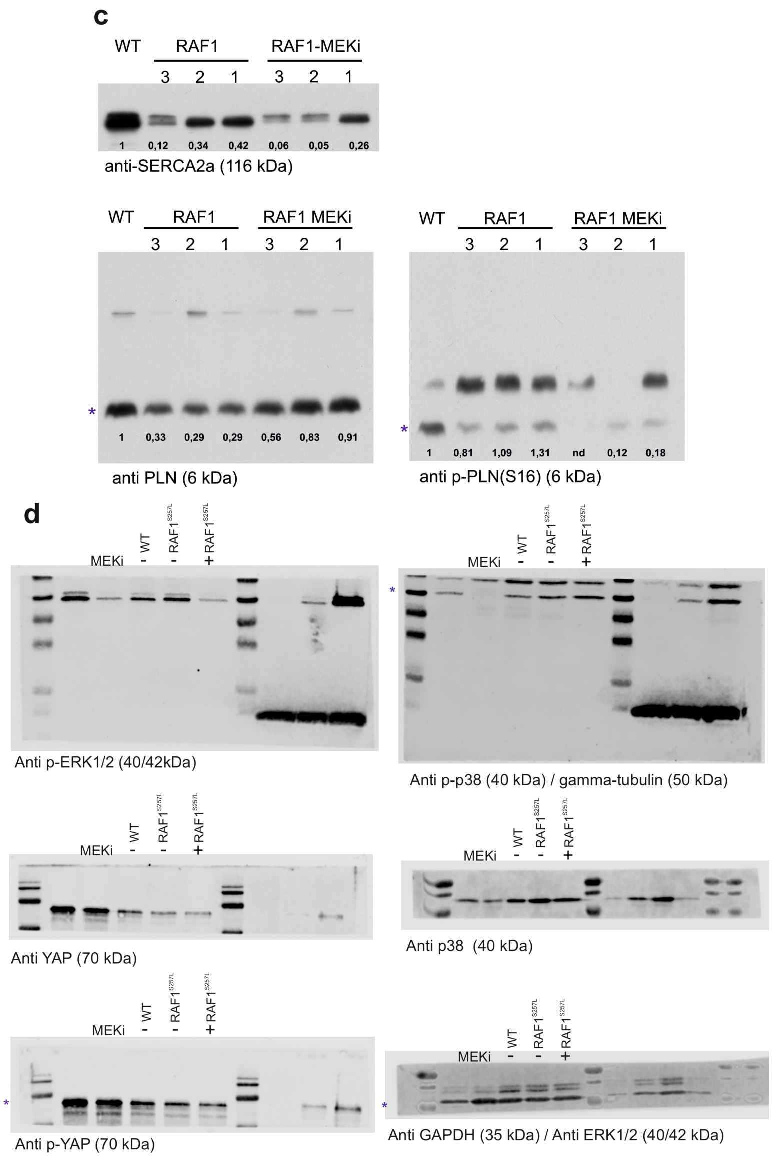

Figure S8. Uncropped and unedited blot/gel images. (a) Immunoprecipitation and quantification of total and p-RAF1^{S259} in WT and RAF1^{S257L} iPSCs (line 1). Membranes were cut at 40 kDa to stain the upper half for CRAF and the lower half for RAS and GAPDH. For more details, see the original legend of Figure 3b. **(b)** Representative immunoblots of p-AKT *vs.* AKT, p-S6K *vs.* S6K, p-RAF1^{259} *vs.* RAF1, p-ERK1/2 *vs.* ERK1/2, p-YAP *vs.* YAP, p-p38 *vs.* p38, and p-JNK *vs.* JNK using cell lysates from WT- and RAF1^{S257}-CBs (d24). SDS gels with 15 chambers were loaded with 7 samples, following by the ladder and 7 more samples. Membranes were cut to stain for more targets. Further, the secondary antibodies were used following the Licor protocol to detect 2 targets on one membrane (700 and 800 nm wavelength). Asterisks (*) indicate the corresponding protein bands if more than one band is present. For more details, see the original legend of Figure 3d. **(c)** Immunoblot analysis of SERCA2, PLN, and p-PLN^{Ser16} in CBs at d24. Asterisks (*) indicate the corresponding protein bands if more than one band is present. For more details, see the original legend of Figure 6e. **(d)** Western blots analysis of the mentioned signaling molecules, p-ERK/ERK, p-YAP/YAP, and p-p38/p38. Asterisks (*) indicate the corresponding protein bands if more than one band is present. For more details, see the original legend of Figure S5b.
