# Supplementary material for: Molecular and cellular evidence for the impact of a hypertrophic cardiomyopathy-associated RAF1 variant on the structure and function of contractile machinery in bioartificial cardiac tissues
Source: Commun Biol. 2023 Jun 21;6:657. doi: 10.1038/s42003-023-05013-8 (PMC10284840; doi:10.1038/s42003-023-05013-8)
Supplement: Supplementary file 5 — Reporting Summary [file 42003_2023_5013_MOESM5_ESM.pdf]

nature portfolio

Corresponding author(s): Mohammad R. Ahmadian, George Kensah, Martin Zenker

Last updated by author(s): 15/05/2023

Reporting Summary

Nature Portfolio wishes to improve the reproducibility of the work that we publish. This form provides structure for consistency and transparency in reporting. For further information on Nature Portfolio policies, see our Editorial Policies and the Editorial Policy Checklist.

Statistics

For all statistical analyses, confirm that the following items are present in the figure legend, table legend, main text, or Methods section.

n/a	Confirmed	
☐	☒	The exact sample size (*n*) for each experimental group/condition, given as a discrete number and unit of measurement
☐	☒	A statement on whether measurements were taken from distinct samples or whether the same sample was measured repeatedly
☐	☒	The statistical test(s) used AND whether they are one- or two-sided<br>*Only common tests should be described solely by name; describe more complex techniques in the Methods section.*
☐	☒	A description of all covariates tested
☐	☒	A description of any assumptions or corrections, such as tests of normality and adjustment for multiple comparisons
☐	☒	A full description of the statistical parameters including central tendency (e.g. means) or other basic estimates (e.g. regression coefficient) AND variation (e.g. standard deviation) or associated estimates of uncertainty (e.g. confidence intervals)
☐	☒	For null hypothesis testing, the test statistic (e.g. *F*, *t*, *r*) with confidence intervals, effect sizes, degrees of freedom and *P* value noted<br>*Give P values as exact values whenever suitable.*
☒	☐	For Bayesian analysis, information on the choice of priors and Markov chain Monte Carlo settings
☒	☐	For hierarchical and complex designs, identification of the appropriate level for tests and full reporting of outcomes
☒	☐	Estimates of effect sizes (e.g. Cohen's *d*, Pearson's *r*), indicating how they were calculated

Our web collection on statistics for biologists contains articles on many of the points above.

Software and code

Policy information about availability of computer code

Data collection	Applied Biosystems 7500 Real-Time PCR: 7500 SDS v1.5.1; Flow cytometry: Diva v6.1.2 (BD Biosciences); TEM: Hitachi H-7100, Karyotype: IKAROS; Western: Odyssey-FC (LI-COR); Confocal microscopy: Zeiss LSM 880 Airyscan.
Data analysis	Microsoft-Excel 2016, LSM Image, ZEN 3.2 (blue edition) by Carl Zeiss AG, Image J, Graphpad Prisim 8, FlowJo, IKAROS, IonWizard, Image Studio lite 5.2 (LI-COR)

For manuscripts utilizing custom algorithms or software that are central to the research but not yet described in published literature, software must be made available to editors and reviewers. We strongly encourage code deposition in a community repository (e.g. GitHub). See the Nature Portfolio guidelines for submitting code & software for further information.

Data

Policy information about availability of data

All manuscripts must include a data availability statement. This statement should provide the following information, where applicable:

- Accession codes, unique identifiers, or web links for publicly available datasets
- A description of any restrictions on data availability
- For clinical datasets or third party data, please ensure that the statement adheres to our policy

Data sharing not applicable to this article as no datasets were generated or analysed during the current study.

Human research participants

Policy information about studies involving human research participants and Sex and Gender in Research.

Reporting on sex and gender	We did not perform the population study according to the sex and gender.
Population characteristics	*Describe the covariate-relevant population characteristics of the human research participants (e.g. age, genotypic information, past and current diagnosis and treatment categories). If you filled out the behavioural & social sciences study design questions and have nothing to add here, write "See above."*
Recruitment	*Describe how participants were recruited. Outline any potential self-selection bias or other biases that may be present and how these are likely to impact results.*
Ethics oversight	Justus-Liebig-University Giessen, Germany: AZ258/16; Otto von Guericke University Medical Center Magdeburg: 173/14; University Medical Center Göttingen: 10/9/15

Note that full information on the approval of the study protocol must also be provided in the manuscript.

Field-specific reporting

Please select the one below that is the best fit for your research. If you are not sure, read the appropriate sections before making your selection.

☒ Life sciences ☐ Behavioural & social sciences ☐ Ecological, evolutionary & environmental sciences

For a reference copy of the document with all sections, see nature.com/documents/nr-reporting-summary-flat.pdf

Life sciences study design

All studies must disclose on these points even when the disclosure is negative.

Sample size	For the experiments performed throughout the manuscript, maximum possible number of samples acquired during the long stretch of the differentiation protocols were used for the best attainable conclusive and explicit interpretation of the experimental results, and the details of the sample numbers and the suitable analysis methods that were used for each individual experiment is mentioned in the corresponding figure legend of that experiment.
Data exclusions	All analyzed data and final results for both iPSC clones were included into the manuscript and the supplemental information.
Replication	Considering the cellular differentiation intervals of approximately 50-60 days for each cardiac differentiation of the corresponding iPSC clone, the general interval for repeating each experiment was from 2 to 3 months. The obtained results for imaging, qPCR, western blotting and functional analysis were from more than 5 to 6 independent cardiac differentiations for each iPSC clone. However, for the experiments performed on the patient biopsy sample, due to the ethical regulations, the experiments were performed using the sample available from one of the patients through the completion of ethical steps and consent form fulfilment.
Randomization	*Describe how samples/organisms/participants were allocated into experimental groups. If allocation was not random, describe how covariates were controlled OR if this is not relevant to your study, explain why.*
Blinding	Entire electron microscopy experiments including processing and imaging of the samples and the data analysis steps were performed in a blind way.

Behavioural & social sciences study design

All studies must disclose on these points even when the disclosure is negative.

Study description	*Briefly describe the study type including whether data are quantitative, qualitative, or mixed-methods (e.g. qualitative cross-sectional, quantitative experimental, mixed-methods case study).*
Research sample	*State the research sample (e.g. Harvard university undergraduates, villagers in rural India) and provide relevant demographic information (e.g. age, sex) and indicate whether the sample is representative. Provide a rationale for the study sample chosen. For studies involving existing datasets, please describe the dataset and source.*
Sampling strategy	*Describe the sampling procedure (e.g. random, snowball, stratified, convenience). Describe the statistical methods that were used to predetermine sample size OR if no sample-size calculation was performed, describe how sample sizes were chosen and provide a rationale for why these sample sizes are sufficient. For qualitative data, please indicate whether data saturation was considered, and what criteria were used to decide that no further sampling was needed.*

Data collection	*Provide details about the data collection procedure, including the instruments or devices used to record the data (e.g. pen and paper, computer, eye tracker, video or audio equipment) whether anyone was present besides the participant(s) and the researcher, and whether the researcher was blind to experimental condition and/or the study hypothesis during data collection.*
Timing	*Indicate the start and stop dates of data collection. If there is a gap between collection periods, state the dates for each sample cohort.*
Data exclusions	*If no data were excluded from the analyses, state so OR if data were excluded, provide the exact number of exclusions and the rationale behind them, indicating whether exclusion criteria were pre-established.*
Non-participation	*State how many participants dropped out/declined participation and the reason(s) given OR provide response rate OR state that no participants dropped out/declined participation.*
Randomization	*If participants were not allocated into experimental groups, state so OR describe how participants were allocated to groups, and if allocation was not random, describe how covariates were controlled.*

Ecological, evolutionary & environmental sciences study design

All studies must disclose on these points even when the disclosure is negative.

Study description	*Briefly describe the study. For quantitative data include treatment factors and interactions, design structure (e.g. factorial, nested, hierarchical), nature and number of experimental units and replicates.*
Research sample	*Describe the research sample (e.g. a group of tagged Passer domesticus, all Stenocereus thurberi within Organ Pipe Cactus National Monument), and provide a rationale for the sample choice. When relevant, describe the organism taxa, source, sex, age range and any manipulations. State what population the sample is meant to represent when applicable. For studies involving existing datasets, describe the data and its source.*
Sampling strategy	*Note the sampling procedure. Describe the statistical methods that were used to predetermine sample size OR if no sample-size calculation was performed, describe how sample sizes were chosen and provide a rationale for why these sample sizes are sufficient.*
Data collection	*Describe the data collection procedure, including who recorded the data and how.*
Timing and spatial scale	*Indicate the start and stop dates of data collection, noting the frequency and periodicity of sampling and providing a rationale for these choices. If there is a gap between collection periods, state the dates for each sample cohort. Specify the spatial scale from which the data are taken*
Data exclusions	*If no data were excluded from the analyses, state so OR if data were excluded, describe the exclusions and the rationale behind them, indicating whether exclusion criteria were pre-established.*
Reproducibility	*Describe the measures taken to verify the reproducibility of experimental findings. For each experiment, note whether any attempts to repeat the experiment failed OR state that all attempts to repeat the experiment were successful.*
Randomization	*Describe how samples/organisms/participants were allocated into groups. If allocation was not random, describe how covariates were controlled. If this is not relevant to your study, explain why.*
Blinding	*Describe the extent of blinding used during data acquisition and analysis. If blinding was not possible, describe why OR explain why blinding was not relevant to your study.*

Did the study involve field work? ☐ Yes ☐ No

Field work, collection and transport

Field conditions	*Describe the study conditions for field work, providing relevant parameters (e.g. temperature, rainfall).*
Location	*State the location of the sampling or experiment, providing relevant parameters (e.g. latitude and longitude, elevation, water depth).*
Access & import/export	*Describe the efforts you have made to access habitats and to collect and import/export your samples in a responsible manner and in compliance with local, national and international laws, noting any permits that were obtained (give the name of the issuing authority, the date of issue, and any identifying information).*
Disturbance	*Describe any disturbance caused by the study and how it was minimized.*

Reporting for specific materials, systems and methods

We require information from authors about some types of materials, experimental systems and methods used in many studies. Here, indicate whether each material, system or method listed is relevant to your study. If you are not sure if a list item applies to your research, read the appropriate section before selecting a response.

Materials & experimental systems

n/a	Involved in the study
☐	☒ Antibodies
☐	☒ Eukaryotic cell lines
☒	☐ Palaeontology and archaeology
☒	☐ Animals and other organisms
☒	☐ Clinical data
☒	☐ Dual use research of concern

Methods

n/a	Involved in the study
☒	☐ ChIP-seq
☐	☒ Flow cytometry
☒	☐ MRI-based neuroimaging

Antibodies

Antibodies used

Santa Cruz; anti-OCT3/4 #sc-5279; anti-Nkx2.5#sc-14033
Thermo Scientific; anti-cardiac troponin t #MA5-12960; anti-TUBB3 #MA1-118
Abcam; anti-Tra-1-60 #ab16288; anti-RAF1#AB181115, anti-phospho-RAF1 S259 #ab173539; anti-troponin I#Ab47003
Synaptic Systems; anti-Myosin light chain 2 V #310111;
DSHB; anti-SSEA4 #MC-813-70;
Sigma-Aldrich; anti- γ -tubulin #T5326, anti-alpha-actinin #A7811
Cell Signaling; anti-phospho-ERK1/2 T202/T204 #9106, Anti-phospho-AKT S473#4060, Anti-phospho-AKT T308#2965, anti-phospho-YAP Ser127#4911, anti-YAP#4912, anti-JNK#9252, anti-phospho-JNK Thr183/Tyr185#9251, anti-S6K#2708, anti-phospho-S6K Thr389#9205, anti-phospho-p38 Thr180/Tyr182#9211, anti-p38#8690, anti-ATP2A2/SERCA2#4388
R&D Systems; anti-Sox17#AF1924
Agilent Technologies; anti-desmin#M076029-2
DAKO; anti-SMA Clone 1A4#M0851

Validation

The antibodies used in this study have been previously used by Nakhaei-Rad et al 2016, Haghighi et al 2018 and Dahlmann J, 2018 (supplementary table 2).
The anti-RAF1# AB181115 was validated using a knock-out line.

Eukaryotic cell lines

Policy information about cell lines and Sex and Gender in Research

Cell line source(s)

We only had two human participant to obtain and generate patient-specific induced pluripotent stem cell (iPSC) lines. Blood sample and dermal fibroblasts were obtained by skin biopsy from two unrelated female patients with Noonan syndrome carrying the heterozygous substitution c.770C>T in exon 7 of RAF1 under protocols concerning research with biomaterials approved by the institution’s ethics committees (Justus-Liebig-University Giessen, Germany: AZ258/16; Otto von Guericke University Medical Center Magdeburg: 173/14; University Medical Center Göttingen: 10/9/15). Research with biomaterials was approved by the ethical committee of the Justus Liebig University of Giessen. Written informed consent was given by both parents of both of the individuals. One human iPSC line that was used in this study as a control, was generated from the human foreskin fibroblast-1 (HFF-1) cell line (ATCC).

Authentication

We had sequenced all Wild-type and mutant iPSCs for RAF1 mutation.

Mycoplasma contamination

Hereby, we confirm that we checked all iPSC lines for mycoplasma and all tested negative.

Commonly misidentified lines
(See ICLAC register)

Name any commonly misidentified cell lines used in the study and provide a rationale for their use.

Flow Cytometry

Plots

Confirm that:

- [ ] The axis labels state the marker and fluorochrome used (e.g. CD4-FITC).
- [ ] The axis scales are clearly visible. Include numbers along axes only for bottom left plot of group (a 'group' is an analysis of identical markers).
- [ ] All plots are contour plots with outliers or pseudocolor plots.
- [x] A numerical value for number of cells or percentage (with statistics) is provided.

Methodology

Sample preparation	Described in supplementary method.
Instrument	BD Accuri C6 flow cytometer
Software	FlowJo
Cell population abundance	Counting at least 10000 single cells.
Gating strategy	Gates and regions were selected around populations of cells with common characteristics (forward scatter, side scatter and marker expression)

- [ ] Tick this box to confirm that a figure exemplifying the gating strategy is provided in the Supplementary Information.
